# The Feasibility of All-Blastocyst-Culture and Single Blastocyst Transfer Strategy in Elderly Women: A Retrospective Analysis

**DOI:** 10.1155/2020/5634147

**Published:** 2020-05-13

**Authors:** Weijue Su, Jialing Xu, Samuel Kofi Arhin, Chang Liu, Junzhao Zhao, Xiaosheng Lu

**Affiliations:** Reproductive Medicine Center, Department of Obstetrics and Gynecology, The Second Affiliated Hospital and Yuying Children's Hospital of Wenzhou Medical University, Wenzhou, Zhejiang 325027, China

## Abstract

**Objective:**

To investigate the feasibility and clinical outcome of the all-blastocyst-culture and single blastocyst transfer strategy in women aged ≥35 years.

**Methods:**

A retrospective analysis of patients aged ≥35 years undergoing IVF/ICSI was performed from January 2017 to April 2019 in the reproductive center of the Second Affiliated Hospital of Wenzhou Medical University. A total of 155 cases treated with ovarian hyperstimulation by prolonged protocol and implemented single (84 cases) or double (71 cases) blastocyst transfer were collected. Then, patients were further divided into <38 yr. group and ≥38 yr. group, and the laboratory and clinical outcomes were compared between the groups.

**Results:**

The double-blastocyst-transfer (DBT) group showed higher clinical pregnancy rate and multiple pregnancy rate and lower neonatal birth weight than those in the single-blastocyst-transfer (SBT) group (*P* < 0.05). However, there were no statistically significant differences between the groups in the embryo implantation rate, biochemical pregnancy rate, miscarriage rate, preterm delivery rate, and term birth rate. For patients < 38 yr., SBT significantly reduced the multiple pregnancy rate and increased the neonate birth weight without significant reduction in the clinical pregnancy rate. While in the ≥38 yr. group, there are no differences in pregnancy outcomes between SBT and DBT. Logistic regression analysis showed that the number of MII oocytes was positively correlated with the live birth rate (OR = 1.18) and negatively correlated with the miscarriage rate (OR = 0.844), suggesting that elderly patients with relatively normal ovarian reserve would obtain better prospect in pregnancy. The number of fetal heart beat in pregnancy was negatively correlated with the live birth rate (OR = 0.322) and positively correlated with the preterm birth rate (OR = 7.16).

**Conclusion:**

The strategy of all-blastocyst-culture and single blastocyst transfer is feasible, safe, and effective for elderly patients with normal ovarian reserve, which would reduce the multiple pregnancy rate.

## 1. Introduction

Currently, the proportion of women delaying childbearing has rapidly increased [1, 2]. Advanced maternal age (≥35 yr.) is associated with a decline in both ovarian reserve and oocyte competence [3], resulting in a decrease of IVF/ICSI pregnancy rate. Although the efficiency and safety of all-blastocyst-culture and single blastocyst transfer (SBT) have been increasingly recognized [4] in patients < 35 yr., information about this protocol has been rarely reported in elderly women (≥35 yr.). In this study, we discussed the feasibility of all-blastocyst-culture and SBT in women ≥ 35 yr. with normal ovarian reserve.

## 2. Data and Methods

### 2.1. Inclusion and Exclusion Criteria

The clinical data of 155 patients undergoing ovarian hyperstimulation from January 2017 to April 2019 in the reproductive center of the Second Affiliated Hospital of Wenzhou Medical University were retrospectively analyzed. The inclusion criteria of this study were (1) 35 years < age ≤ 43 years, (2) no contraindications to IVF/ICSI in routine preoperative physical examination, (3) body mass index (BMI) < 27 kg/m^2^, (4) antral follicle count (AFC) ≥ 5, and (5) basal follicle − stimulating hormone (b − FSH) ≤ 12 IU/L. The exclusion criteria include (1) patients whose husbands have chromosomal abnormality, azoospermia, severe oligospermia, and asthenozoospermia; (2) endometriosis; (3) adenomyosis; (4) endometrial fibroids; (5) uterine cavity adhesion; (6) endometrial polyps; (7) hydrosalpinx; (8) recurrent miscarriage; (9) chromosomal abnormalities in either of the couples; and (10) previous IVF/ICSI treatment failed >2 times.

### 2.2. Controlled Ovarian Hyperstimulation (COH): The Prolonged Protocol

All patients were subcutaneously injected with gonadotropin-releasing hormone agonist (GnRH-a) 3.75 mg at the 3^rd^-5^th^ days of their menstrual cycle. After 35-38 days, gonadotropin (Gn) such as recombinant follicle-stimulating hormone (rFSH, 150-225 IU/day, Merck Serono Co. Ltd., Swiss) was performed for ovary stimulation. And the dose was adjusted according to patient's age, AFC, BMI, b-FSH level, and the follicle number/size. Once the diameter of the largest follicle reached 12 mm, high-purity human menopause gonadotropin (HP-HMG, 0-150 IU/day, Merck Co. Ltd., Germany) was added. If there was still no advantageous follicle on the 10^th^ day of COH, treatment should be cancelled. When there are 2-3 follicles developing larger than 16 mm in diameter, or the diameter of the largest follicle ≥ 18 mm, intramuscular injection of human chorionic gonadotropin (hCG, 10000 IU, LIZHU Pharmaceutical Factory, China) at 21:00 was executed (trigger).

### 2.3. Oocyte Retrieve and Incubation Process

Ultrasound-guided transvaginal oocyte retrieval was performed 34-36 hours after trigger. The retrieved oocytes were fertilized by IVF/ICSI 4-6 h after oocyte retrieval. Then, embryos were incubated at 37°C under a humidified gas phase of a mixture of 4% O_2_, 6% CO_2_, and 90% N_2_. And the development of embryos was firstly evaluated 16-18 hours later. Normal fertilization was confirmed by the presence of two polar bodies and two pronuclear 16–18 h after insemination. The number of blastomeres, blastomere size, and the proportion of fragments were assessed in a cleavage-stage embryo. For morulae-stage embryos, the proportion of compaction was evaluated. On the 5^th^ day, blastocyst formed. Blastocyst scoring was graded according to the morphology including the expansion stage of blastocyst cavity expansion, density, cell number of inner cell mass, and the cohesion as well as regularity of trophectoderm (Alpha Scientists in Reproductive Medicine and ESHRE Special Interest Group of Embryology, 2011) [5].

### 2.4. Embryo Transfer and Luteal Support

According to the guidelines from the American Society for Reproductive Medicine (ASRM) [6] (two blastocysts can be transferred for elderly patients, but a single good-quality blastocyst is the preferred option), 1 or 2 blastocysts were transferred under the guidance of B-ultrasound. The number of embryos transferred was determined by both the physician and the couple, depending on the patient's willingness, the outcome of previous IVF/ICSI cycles, the number of blastocysts available, and the quality of embryos. Luteal phase support was implemented with 200 mg orally micronized progesterone per day (Utrogestan; Capsugel, Besins Manufacturing Belgium, Bruxelles, Belgium) and 90 mg progesterone gel via vagina per day (Crinone; Merck Serono, Hertfordshire, United Kingdom) from the day of oocyte retrieval.

### 2.5. Follow-Up

Serum hCG levels were measured 12 days after embryo transfer to determine pregnancy. Another 2 weeks later, clinical pregnancy was defined as the presence of pregnancy sac and fetal heart beat under transvaginal ultrasound. Besides, hCG positive without pregnancy sac 45 days after embryo transfer was defined as biochemical pregnancy. Miscarriage criterion was defined as embryo loss before 20 gestational weeks, and premature delivery occurred between 28 and 37 gestational weeks. Delivery between 37 and 42 weeks of gestation was term delivery. Neonate birth weight < 2500 g was considered as low-weight infants, while ≥4000 g was fetal macrosomia.

### 2.6. Observation Indicators

The patients' background data, clinical and laboratory indicators, and pregnancy rate were compared between the groups. hCG‐positive rate (%) = hCG‐positive cases/total cases × 100; ectopic pregnancy rate (%) = ectopic pregnancy cases/clinical pregnancy cases × 100; clinical pregnancy rate (%) = clinical pregnancy cases/total cases × 100; biochemical pregnancy rate (%) = No.of biochemical pregnancy/No.of hCG‐positive number × 100; multiple pregnancy rate (%) = No.of multiple pregnancy/No.of clinical pregnancy × 100; embryo implantation rate (%) = No.of implanted embryos/No.of total transferred embryos × 100; early miscarriage rate (%) = early miscarriage cases/clinical pregnancy cases × 100.

### 2.7. Statistics

SPSS 24.0 statistical software was used for data analysis. Continuous data was analyzed by the independent samples *t*-test or Mann-Whitney test. The chi-square test and Fisher's exact test were used in data analysis for dichotomous variables. Binomial logistic regression and Hosmer-Lemeshow were used to administrate risk factor analysis. In this research, *P* < 0.05 was considered statistically significant difference.

## 3. Results

### 3.1. Compare between the SBT and DBT Groups

#### 3.1.1. Background Data

A total of 155 cycles were included in this study, including 84 cases in the single-blastocyst-transfer (SBT) group and 71 cases in the double-blastocyst-transfer (DBT) group. There were no statistically significant differences in BMI, infertility duration, fertilization, baseline hormone concentrations, and AFC between the two groups (*P* > 0.05). The patients' age in the SBT group was younger than that in the DBT group (*P* < 0.05) (Table 1).

#### 3.1.2. Clinical and Laboratory Indicators of SBT and DBT

There were no statistically differences in endometrium thickness on trigger day, days of Gn, dose of total Gn, No. of oocytes retrieved, rate of fertilization, No. of transferrable embryos on D3, and No. of embryos on D5 between the two groups (*P* > 0.05), as shown in Table 2. The SBT group showed higher No. of good-quality D5 embryos and rate of good-quality embryos on D5 (*P* < 0.01), as shown in Table 2.

#### 3.1.3. Pregnancy Outcomes of SBT and DBT

The rate of clinical pregnancy in the SBT group was 46.4%, compared with 56.3% in the DBT group without statistical difference. There was no statistically difference in the miscarriage rate, biochemical pregnancy rate, ectopic pregnancy rate, embryo implantation rate, premature birth rate, term birth rate, live birth rate, fetal macrosomia rate, and low-weight infant rate between the two groups. Due to the decline of the embryo number transferred, the multiple pregnancy rate of the SBT group was significantly lower than that the DBT group (0 vs. 22.5%), leading to a lower preterm birth rate (although no statistical difference), significantly increased neonatal birth weight (*P* < 0.05), as shown in Table 3.

#### 3.1.4. Risk Factors of Pregnancy Outcome in Elderly Women Undergoing IVF/ICSI

Logistic regression showed that the risk of miscarriage increased with maternal age (*P* < 0.05, OR: 1.327, 95% CI: 1.01-1.75). After adjusting endometrium thickness on trigger day, infertility years, and age, the number of oocytes retrieved and fetal heart beats became independent factors in predicting live birth (*P* < 0.05, OR: 1.18, 95% CI: 1.01-1.38 and OR: 0.322, 95% CI: 0.11-0.95, respectively), as the number of MII oocytes increased, the probability of live birth increased. And the number of fetal heart beats made the reverse influence. After adjusting BMI, the number of fetal heart beats was the risk factor for predicting preterm birth (*P* < 0.05, OR: 7.16, 95% CI: 1.63-31.45), and the risk of having two fetal heart beats was 7.16 times than that of the single one (Table 4).

### 3.2. SBT in Different Age of Elderly Women

The above study showed that patients in the SBT group were younger than those in the DBT group, suggesting that younger patients were more likely to accept SBT. Thus, according to an important age node of IVF/ICSI success rate in women ≥ 35 yr., we divided the patients into groups of <38 yr. and ≥38 yr. The pregnancy outcomes of SBT and DBT were compared inside the different age groups.

#### 3.2.1. SBT vs. DBT in Patients Aged <38 yr.

A total of 98 patients aged 35-38 yr. were included in this study, including 59 patients choosing SBT and the other 39 having DBT. There were no statistically differences in infertility duration, BMI, fertilization methods, baseline hormone concentrations, and AFC between the two groups (*P* > 0.05), as shown in Table 5.

The number of oocytes retrieved, MII oocytes, D5 embryos, good-quality D5 embryos, and the rate of good-quality D5 embryos in the SBT group were all higher than those in the DBT group (*P* < 0.05). As a fact, patients with better laboratory outcomes were more likely to choose SBT (Table 6).

Due to the low number of embryos transferred in the SBT group, the number of fetal heart beats was significantly lower than that in the DBT group, resulting in 0% multiple pregnancy rate compared with 38.1% in the DBT group. Multiple births led to the newborns' weight of the DBT group were significantly lower than that of the SBT group (Table 7).

#### 3.2.2. SBT vs. DBT in Patients Aged ≥38 yr.

A total of 57 results of cycles were collected, which included 25 SBT and 32 DBT cases. There were no statistically differences in infertility duration, BMI, fertilization methods, baseline hormone concentrations, and AFC between the two groups (*P* > 0.05, Table 8).

The endometrium thickness of the DBT group was thicker than that of the SBT group at the trigger day, which may be more conducive to embryo implantation and increased the pregnancy rate. Besides, there was no difference between the two groups in the laboratory indicators such as the days of Gn, the dose of total Gn, number of oocytes retrieved, number of MII oocytes, fertilization rate, number/rate of D5 embryos, and number/rate of good-quality D5 embryos (*P* > 0.05), as shown in Table 9. Due to the low proportion of patients ≥ 38 yr. with normal ovarian reserve, more cases need to be collected for a longer time period in any further research.

Furthermore, there was no difference in pregnancy outcomes between the SBT and DBT strategy (*P* > 0.05), in spite of the DBT group having the thicker endometrium. In this case, SBT may provide a positive effect in elderly patients (Table 10).

## 4. Discussion

In recent years, advances in assisted reproductive technologies (ART), particularly in embryo culture and cryopreservation, have improved rapidly and resulted in the improvement of live birth rate. Based on the increase of success rate, there has been a shift in the practice of ART from the primary goal of achieving live birth to optimize maternal and neonatal safety. Meanwhile, multiple pregnancy is considered to be the most significant adverse event with the increase of maternal and neonatal morbidity rate [7]. Limiting the number of transferred embryos, specifically adopting the practice of elective single embryo transfer (eSET), has been shown to be the most effective strategy to decrease the risk of multiple pregnancy [8]. Due to the high risk of obstetrical and neonatal complications associated with advanced maternal age, eSET is particularly meaningful in elderly women undergoing ART [9].

Currently, for women ≥ 35 yr., there is no treatment clearly improving the quality of the aging gamete or embryo; hence, it would be a most effective choice to adjust the appropriate embryo transfer strategy to maximize the pregnancy rate. Reliable embryo selection strategy is pivotal to increase IVF efficiency (i.e., lower miscarriage rate, higher implantation rate, and live birth rate) and safety (i.e., lower maternal and neonatal complication). Single embryo transfer (SET) policy, in turn, also minimizes the risk for multiple gestations.

The development of blastocyst incubation technology is progressing rapidly in recent years. In the practice of young patients (<35 yr.), compared to the cleavage embryo transfer, blastocyst transfer improved the embryo utilization rate [10] and live birth rate [11] and reduced the ectopic pregnancy rate [12]. It was reported that 59% of good-quality D3 embryos contain abnormal chromosomes, while only 35% of good-quality D5 blastocysts were aneuploid [13]. Researches have even found that 80%~90% of the D3 embryo implantation failure might derive from the high morphological score but low actual quality [14]. Blastocyst culture may eliminate the D3 embryos with a high morphological score but low develop potential to be transferred [15].

However, blastocyst culture requires high laboratorial environment and technic [16]. Women ≥ 35 years old experience a dramatic increase in embryo aneuploidy rate from a 30% baseline production up to 90% in their late 40s prior to the menopause [17, 18]. Specifically, the chance of producing a chromosomally normal blastocyst might be even lower than 5% in women older than 43 years [19, 20]. For the elderly women, all-blastocyst-incubation strategy implies the risk of zero blastocyst formation and embryo transfer cancel. This is a big blow for the elderly couple struggling to have a child.

The risk of the zero-blastocyst formation had expressed pressure on both elderly couples and clinicians considering all-blastocyst-culture. The age-related decline in ovarian reserve not only reduces the chances of getting a blastocyst but also reduces the rate of embryo implantation and pregnancy [21] and makes it difficult for patients and doctors to consider SBT. Therefore, the clinical outcomes of SBT and DBT have always been a research hot spot. Most studies have just focused on younger patients (<35 years old). Few literatures have pay attention to all-blastocyst-culture and SBT in elderly women ≥ 35 yr. To the best of our knowledge, the feasibility and outcomes of single blastocyst transfer (SBT) in elderly patients remain unknown. In this study, we chose women aged ≥35 yr. with normal ovarian reserve and compared the laboratorial and clinical outcomes of SBT and DBT strategy.

Our investigation showed that among women ≥ 35 yr. with normal ovarian reserve, the inclined to choose DBT increases with patient' age and the risk of miscarriage rises. Besides, DBT increased the pregnancy rate and live birth rate compared with SBT and also increased the multiple pregnancy rate, the proportion of low-weight infants, and the risk of preterm birth. The age of women was higher in undergoing DBT than SBT. That was to say, in our center, elder couples were more intend to transfer two blastocysts rather than one single embryo. While studies by Tannus et al. [22] had drawn different conclusions that the average age of DBT is similar to SBT. A Canadian study showed that 40% of IVF patients wish to have multiple pregnancies [23], which may be related to the pain of longtime infertility and anxiety over the failure of repeated fertility treatments. However, logistic regression analysis showed that age was a risk factor for miscarriage, but there was no statistical difference in miscarriage rate between DBT and SBT groups in our study. Tannus et al. reached the same conclusion when comparing the miscarriage rate of SBT and DBT [22].

According to the latest guidelines issued by the American Society for Reproductive Medicine (ASRM) [6], elderly patients can choose two blastocyst transfer, but one single good-quality blastocyst is preferred. However, a number of studies have shown that there was no statistical difference in implantation rate between SBT and DBT, but DBT may act better at increasing live birth rate [24, 25]. Whether stratified by age or not, our research did not show any increase in implantation or live birth rate in the DBT group, but the rate of preterm delivery was raised and neonatal birth weight was significantly reduced with the increasing of multiple pregnancy rate. Meta-analysis of 146,008 cases of multiple births showed that multiple pregnancies were associated with high risk of adverse pregnancy outcomes [26]. In addition, several studies indicated that more than 50% of women during twin pregnancy suffered placenta ischemic complications [27], which may be age related [28, 29]. Therefore, it is still worth advocating to carry out SBT in elderly women for reducing multiple pregnancy rate.

However, persuading elderly couples to accept SBT remains a challenge. Only less than a quarter of women were open to SBT [30]. One of the important reasons is that patients consider double embryo transfer would improve the success rate of pregnancy. As the professional information provider, clinicians play a key role in decision-making. Therefore, doctors should repeatedly interpret the negative impact of multiple pregnancy on both maternal and child health and even economic pressure on families to elderly couples before they make the decision of how many embryos to be transferred.

## 5. Conclusion

In conclusion, our data showed that, in women ≥ 35 yr. with normal ovarian reserve, DBT did not increase the implantation rate or live birth rate but rose the rate of preterm delivery and significantly reduced the neonatal birth weight by the increase of multiple pregnancy rate. The risk of all-blastocyst-culture is safe and effective in this certain group of elderly women. In turn, single embryo transfer (SET) policy minimizes the risk for multiple gestations. Thus, we suggest that the strategies of all-blastocyst-culture and single blastocyst transfer are feasible, safe, and effective for elderly patients with normal ovarian reserve.

## Figures and Tables

**Table 1:** Background data of SBT and DBT.

	SBT (*n* = 84)	DBT (*n* = 71)	*P* value
Age	36.8 (35.7, 38.6)	37.6 (35.9, 40.1)	0.019^∗^
Infertility duration (years)	3.9 ± 3.4	3.5 ± 3.1	0.472
BMI (kg/m^2^)	21.9 ± 3.0	21.8 ± 3.0	0.773
AFC	12.9 ± 4.9	12.0 ± 4.3	0.201
Fertilization method (%)			
IVF	80.0 (67/84)	70.4 (50/71)	0.125
ICSI	17.9 (15/84)	21.1 (15/71)	0.478
IVF+ICSI	2.4 (2/84)	8.5 (6/71)	0.092
AMH (ng/mL)	3.0 ± 1.9	3.6 ± 4.1	0.568
Baseline gonadal hormone			
LH (IU/L)	4.3 ± 2.0	3.7 ± 1.6	0.060
FSH (IU/L)	6.9 ± 2.3	6.9 ± 1.8	0.965
E2 (ng/mL)	61.9 ± 49.3	58.3 ± 44.0	0.665
P (nmol/L)	0.52 (0.37, 0.73)	0.51 (0.4, 0.68)	0.402

^∗^ *P* < 0.05.

**Table 2:** Clinical and laboratory indicators of SBT and DBT.

	SBT (*n* = 84)	DBT (*n* = 71)	*P* value
Days of Gn	11.4 ± 2.1	11.4 ± 2.1	0.887
Dose of total Gn (IU)	2727.0 ± 785.6	2832.8 ± 784.8	0.405
Endometrium thickness (mm)	10.4 ± 2.9	11.0 ± 2.9	0.220
No. of oocytes retrieved	12.5 (9.25, 18)	12 (8, 15)	0.058
No. of transferrable D3 embryo	5 (3, 7)	5 (3, 6)	0.063
No. of MII oocytes	11.0 (8, 16)	10.0 (8, 13)	0.097
Rate of fertilization (%)	81.9 ± 16.2	83.6 ± 13.1	0.488
No. of D5 embryos	4.0 (3, 5.8)	3.0 (2, 5)	0.102
No. of good-quality D5 embryos	3.0 ± 2.0	2.0 ± 2.0	0.007^∗∗^
Rate of good-quality D5 embryos (%)	73.6 ± 26.7	53.44 ± 34.9	0.001^∗∗^

^∗^ *P* < 0.05, ^∗∗^*P* < 0.01.

**Table 3:** Pregnancy outcomes of SBT and DBT.

	SBT (*n* = 84)	DBT (*n* = 71)	*P* value
No. of fetal heart beats	38	55	0.005^∗∗^
Rate of clinical pregnancy (%)	46.4 (39/84)	56.3 (40/71)	0.219
Rate of chemistry pregnancy (%)	11.9 (10/49)	14.9 (7/47)	0.479
Rate of ectopic pregnancy (%)	2.6 (1/39)	0.0 (0/40)	0.990
Implantation rate (%)	45.2 (38/84)	43.7 (62/142)	0.818
Multiple pregnancy rate (%)	0.0 (0/39)	22.5 (9/40)	0.005^∗∗^
Miscarriage rate (%)	18.0 (7/39)	12.5 (5/40)	0.500
Preterm birth rate (%)	7.7 (3/39)	20.0 (8/40)	0.114
Term delivery rate (%)	71.8 (28/39)	62.5 (25/40)	0.379
Live birth rate (%)	37.0 (31/84)	46.5 (33/71)	0.228
Rate of low-weight infants (%)	6.5 (2/31)	19.0 (8/42)	0.229
Rate of fetal macrosomia (%)	6.5 (2/31)	4.7 (2/42)	0.787
Neonatal birth weight (g)	3210.1 ± 368.5	2989.9 ± 481.6	0.034^∗^

^∗^ *P* < 0.05, ^∗∗^*P* < 0.01.

**Table 4:** Risk factors of pregnancy outcome in elderly women underwent IVF/ICSI.

Predictive and response variables	Beta ± SE	*P* value	OR (95% CI)
Live birth			
Hosmer-Lemeshow test		0.963	
No. of MII oocytes	0.161 ± 0.080	0.044^∗^	1.18 (1.01-1.38)
No. of fetal heart beats	−1.134 ± 0.552	0.040^∗^	0.322 (0.11-0.95)
Miscarriage			
Hosmer-Lemeshow test		0.913	
Age	0.283 ± 0.142	0.046^∗^	1.327 (1.01-1.75)
No. of MII oocytes	−0.170 ± 0.086	0.049^∗^	0.844 (0.71-0.999)
Preterm birth			
Hosmer-Lemeshow test		0.874	
No. of fetal heart beats	1.969 ± 0.755	0.009^∗∗^	7.16 (1.63-31.45)
No. of D5 embryo	0.436 ± 0.211	0.039^∗^	1.56 (1.02-2.34)

^∗^ *P* < 0.05, ^∗∗^*P* < 0.01.

**Table 5:** Background information of patients aged 35-38 yr.

	SBT (*n* = 59)	DBT (*n* = 39)	*P* value
Infertility duration (years)	4.0 ± 3.2	4.0 ± 3.2	0.990
BMI (kg/m^2^)	21.8 ± 3.0	21.4 ± 3.1	0.614
AFC	13.7 ± 4.9	11.2 ± 2.8	0.429
Fertilize method (%)			
IVF	80.0 (46/59)	71.8 (28/39)	0.487
ICSI	20.3 (12/59)	18.0 (7/39)	0.770
IVF+ICSI	1.7 (1/59)	10.3 (4/39)	0.080
Baseline gonadal hormone			
LH (IU/L)	4.6 ± 2.2	4.0 ± 1.7	0.189
FSH (IU/L)	7.0 ± 2.2	7.2 ± 1.8	0.540
E2 (ng/mL)	54.5 ± 31.3	59.4 ± 41.0	0.524
P (nmol/L)	1.2 ± 2.0	0.8 ± 1.2	0.424

**Table 6:** Clinical and laboratory indicators in women aged 35-38 yr.

	SBT (*n* = 59)	DBT (*n* = 39)	*P* value
Days of Gn	11.6 ± 2.1	11.7 ± 2.2	0.720
Dose of total Gn (IU)	2766.4 ± 763.7	2857.7 ± 835.8	0.578
Endometrium thickness (mm)	11.1 ± 2.8	11.2 ± 2.8	0.784
No. of oocytes retrieved	14.0 (10, 18)	12.0 (8, 14)	0.005^∗∗^
No. of MII oocytes	12.0 (9, 17)	11.0 (7, 13)	0.027^∗^
Rate of fertilization (%)	83.1 ± 14.7	82.1 ± 15.4	0.762
No. of D5 embryo	4.0 (3, 7)	3.0 (2, 4)	0.019^∗^
No. of good-quality D5 embryo	3.4 ± 2.3	2.0 ± 2.0	0.002^∗∗^
Rate of good-quality D5 embryo (%)	75.0 (60, 100)	50.0 (0, 80)	0.001^∗∗^

^∗^ *P* < 0.05, ^∗∗^*P* < 0.01.

**Table 7:** Pregnancy outcomes of women aged 35-38 yr.

	SBT (*n* = 59)	DBT (*n* = 39)	*P* value
No. of fetal heart beats	25	33	0.009^∗∗^
Rate of clinical pregnancy (%)	44.1 (26/59)	53.8 (21/39)	0.306
Rate of chemistry pregnancy (%)	25.7 (9/35)	19.2 (5/26)	0.552
Rate of ectopic pregnancy (%)	3.8 (1/26)	0.0 (0/21)	0.553
Implantation rate (%)	44.1 (26/59)	44.9 (35/78)	0.770
Multiple pregnancy rate (%)	0.0 (0/26)	38.1 (8/21)	0.001^∗∗^
Miscarriage rate (%)	15.4 (4/26)	9.5 (2/21)	0.442
Preterm birth rate (%)	14.3 (3/21)	28.6 (6/21)	0.135
Term delivery rate (%)	69.2 (18/26)	61.9 (13/21)	0.598
Live birth rate (%)	35.6 (21/59)	48.7 (19/39)	0.196
Rate of low-weight infants (%)	9.5 (2/21)	35.0 (7/20)	0.092
Rate of fetal macrosomia (%)	9.5 (2/21)	0 (0/27)	0.186
Neonatal birth weight (g)	3219.1 ± 627.8	2832.6 ± 469.6	0.019^∗^

^∗^ *P* < 0.05, ^∗∗^*P* < 0.01.

**Table 8:** Background information of patients aged ≥38 yr.

	SBT (*n* = 25)	DBT (*n* = 32)	*P* value
Infertility duration (years)	3.8 ± 4.0	3.0 ± 3.0	0.409
BMI (kg/m^2^)	22.3 ± 3.0	22.1 ± 2.8	0.858
AFC	11.0 ± 4.4	10.8 ± 3.8	0.812
Fertilize method (%)			
IVF	84.0 (21/25)	71.9 (22/32)	0.187
ICSI	12.0 (3/25)	25.0 (8/32)	0.315
IVF+ICSI	4.0 (1/25)	6.3 (2/32)	1
Baseline gonadal hormone			
LH (IU/L)	3.24 (2.68, 5.07)	3.29 (2.51, 4.41)	0.318
FSH (IU/L)	6.7 ± 2.7	6.5 ± 1.8	0.740
E2 (ng/mL)	79.2 ± 74.4	57.1 ± 48.0	0.198
P (nmol/L)	0.9 ± 2.0	0.8 ± 1.3	0.827

^∗^ *P* < 0.05, ^∗∗^*P* < 0.01.

**Table 9:** Clinical and laboratory indicators in women aged ≥38 yr.

	SBT (25 cases)	DBT (32 cases)	*P* value
Days of Gn	11.1 ± 2.1	11.0 ± 1.9	0.423
Dose of total Gn (IU)	2634 ± 843.9	2802.4 ± 730.0	0.779
Endometrium thickness (mm)	9.1 ± 2.6	10.8 ± 3.2	0.029^∗^
No. of oocytes retrieved	11.6 ± 5.6	12.6 ± 4.5	0.476
No. of MII oocytes	10.4 ± 5.2	11.0 ± 4.1	0.663
Rate of fertilization (%)	83.0 (68.5, 96.5)	86.0 (78, 93.5)	0.158
No. of D5 embryo	3.3 ± 2.1	3.8 ± 2.3	0.424
No. of good-quality D5 embryo	2.2 ± 1.4	2.4 ± 1.9	0.698
Rate of good-quality D5 embryo (%)	74.0 ± 28.6	58.7 ± 30.7	0.060

^∗^ *P* < 0.05, ^∗∗^*P* < 0.01.

**Table 10:** Pregnancy outcomes of SBT and DBT in women aged ≥38 yr.

	SBT (25 cases)	DBT (32 cases)	*P* value
No. of fetal heart beats	13	23	0.194
Rate of clinical pregnancy (%)	52.0 (13/25)	59.4 (19/32)	0.578
Rate of chemistry pregnancy (%)	7.1 (1/14)	9.5 (2/21)	1
Rate of ectopic pregnancy (%)	0 (0/13)	0.0 (0/19)	—
Implantation rate (%)	52.0 (13/25)	42.1 (27/64)	0.403
Multiple pregnancy rate (%)	0.0 (0/13)	5.3 (1/19)	1
Miscarriage rate (%)	23.1 (3/13)	15.8 (3/19)	0.666
Preterm birth rate (%)	0 (0/13)	10.5 (2/19)	0.502
Term delivery rate (%)	40.0 (10/25)	37.5 (12/32)	0.467
Live birth rate (%)	40.0 (10/25)	43.8 (14/32)	0.794
Rate of low-weight infants (%)	0 (0/10)	6.7 (1/15)	1
Rate of fetal macrosomia (%)	10.0 (1/10)	6.7 (1/15)	1
Neonatal birth weight (g)	3125 (2969, 3362.5)	3250 (2900, 3650)	0.439

^∗^ *P* < 0.05, ^∗∗^*P* < 0.01.

## Data Availability

All data are available in the manuscript.
